# Supplementary figures and images for: The Cif proteins from Wolbachia prophage WO modify sperm genome integrity to establish cytoplasmic incompatibility
Source: PLoS Biol. 2022 May 24;20(5):e3001584. doi: 10.1371/journal.pbio.3001584 (PMC9128985; doi:10.1371/journal.pbio.3001584)

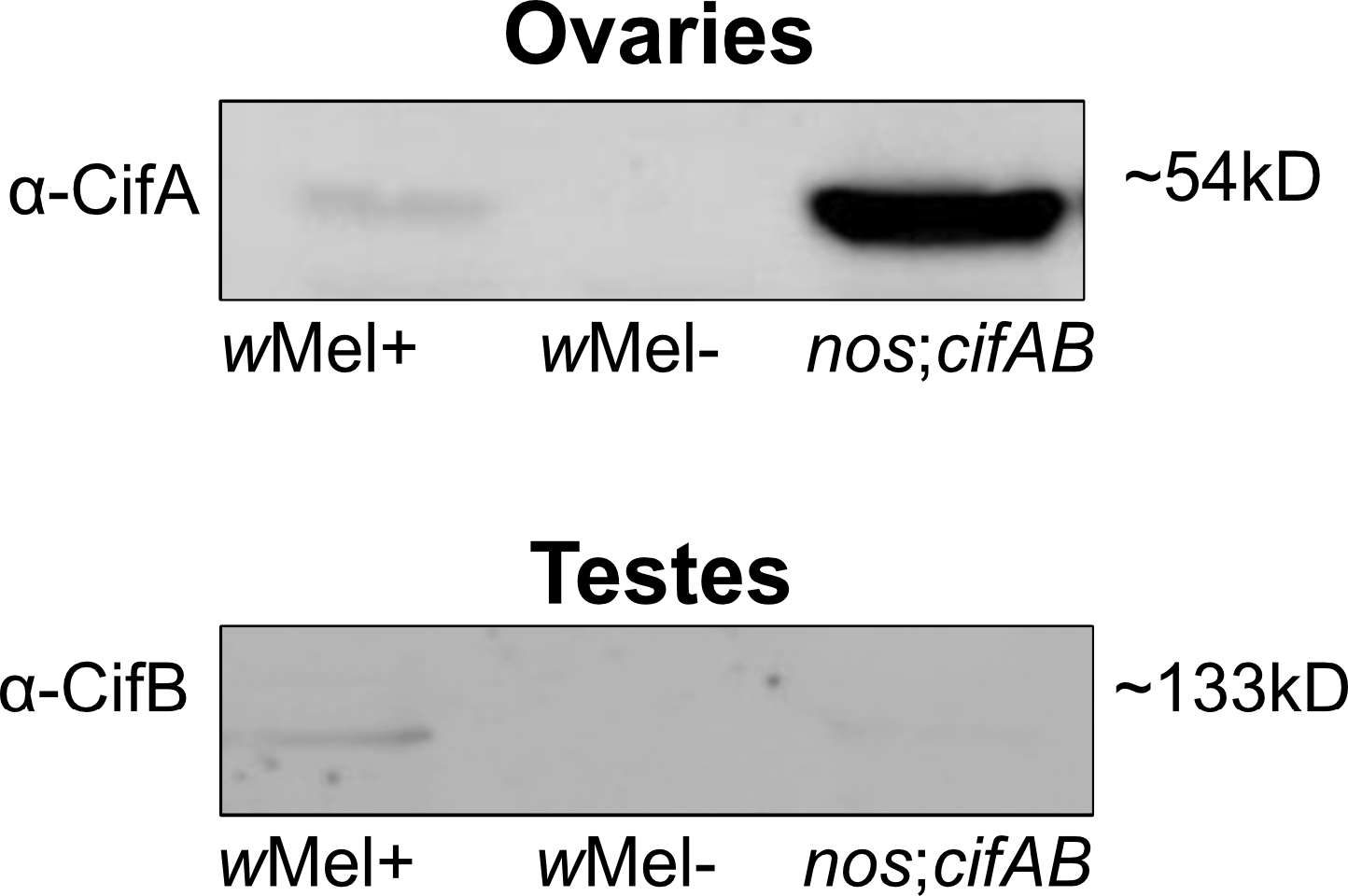

Supplement: S1 Fig — Western blots were run on protein extracted from ovaries (n = 10) of wild-type infected wMel+, uninfected wMel−, cifA transgenic (cifA), and dual cifA;B expressing transgenic lines. The expected size for CifA is approximately 54 kD. Western blots were run using anti-CifB antibody on testes (n = 15) of wild-type infected (+), uninfected (−), and cifA;B transgenic (A;B) lines. Expected CifB size is approximately 133kD. Cifs are absent in wMel− control and present at accurate size in wMel+ and cif expressing lines. (TIF) [file pbio.3001584.s001.tif]

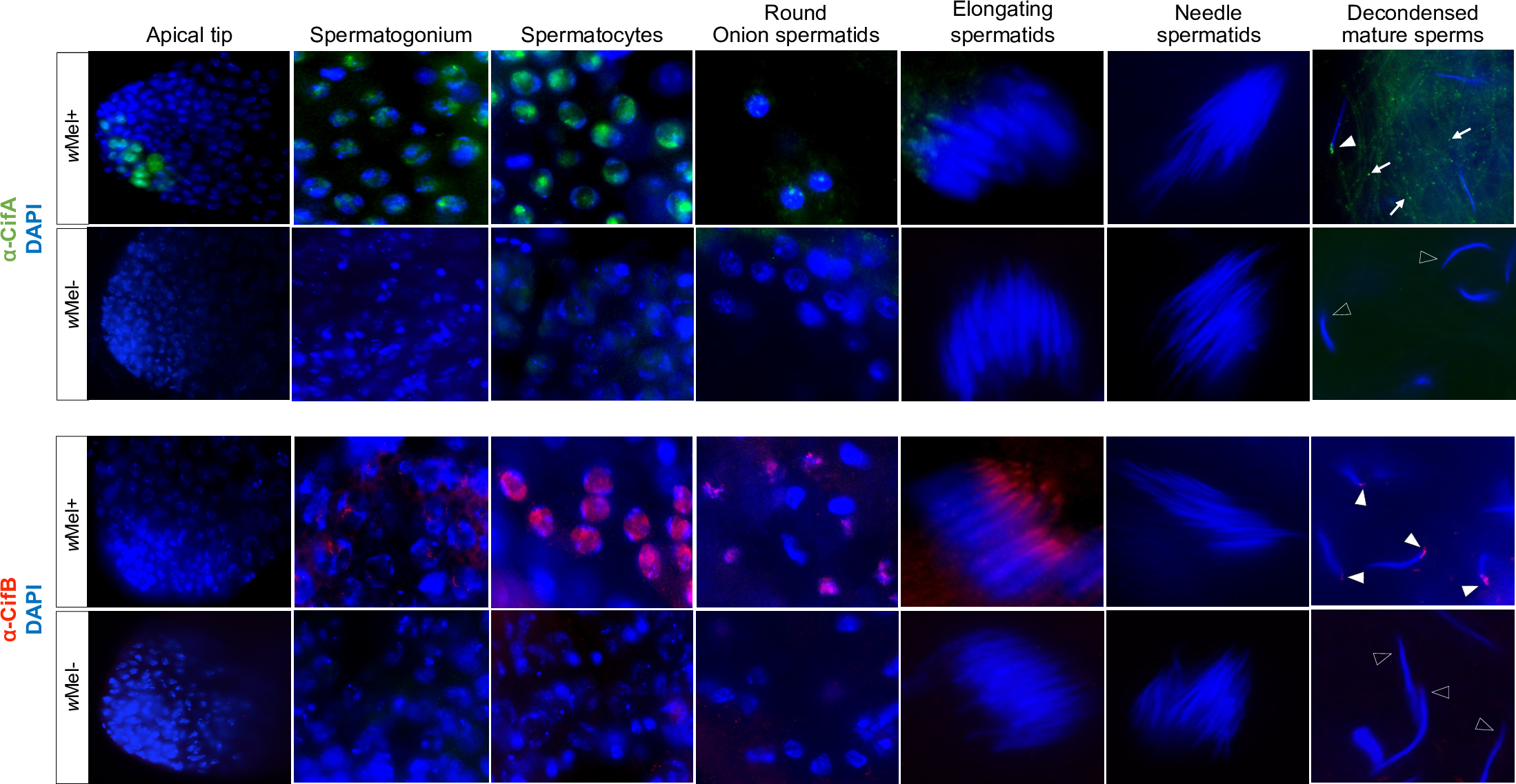

Supplement: S2 Fig — Testes (n = 20) from <8-hour-old males of wild-type wMel+ and wMel− lines were dissected and immunostained to visualize CifA (green) and CifB (red) during sperm morphogenesis. DAPI stain (blue) was used to label nuclei. CifA and CifB localization patterns in wild-type lines are similar to that of transgenic cifAB (Fig 1) and signals are absent in wMel− uninfected control line. The experiment was conducted in parallel to the one shown in Fig 1. (TIF) [file pbio.3001584.s002.tif]

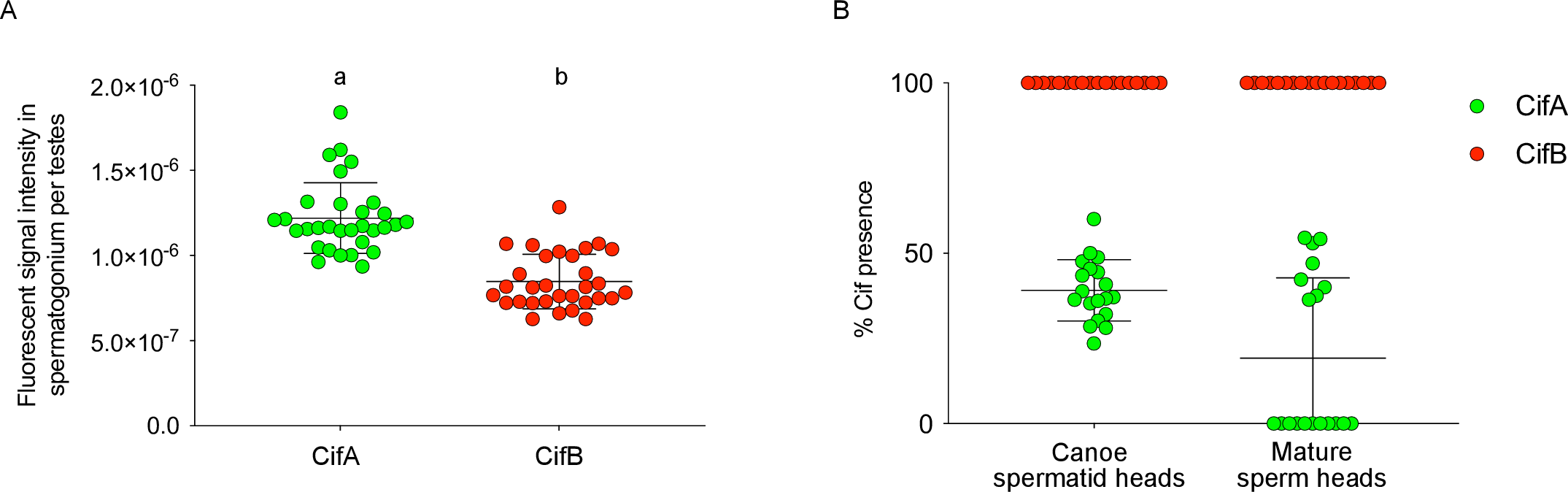

Supplement: S3 Fig — (A) ImageJ-based signal intensity quantification indicates CifA (green) is more abundantly expressed than CifB (red) in the spermatogonium stage of the spermatogenesis. Mean of individual data points with standard deviation is plotted on the graph. Letters indicate statistically significant (p < 0.05) differences as determined by pairwise comparisons based on a Mann–Whitney test. p-Values are reported in S1 Table. (B) In the decondensed mature sperms isolated from seminal vesicles, CifB is present in the acrosomal tip of canoe-shaped spermatids and mature sperm heads, whereas CifA is present in only 40% and 20% of them, respectively. Quantification was performed on the images obtained in Fig 1 data. Each dot represents percentage of Cifs present in spermatids or mature sperms per testes examined. Raw data underlying this figure can be found in S1 Data file. (TIF) [file pbio.3001584.s003.tif]

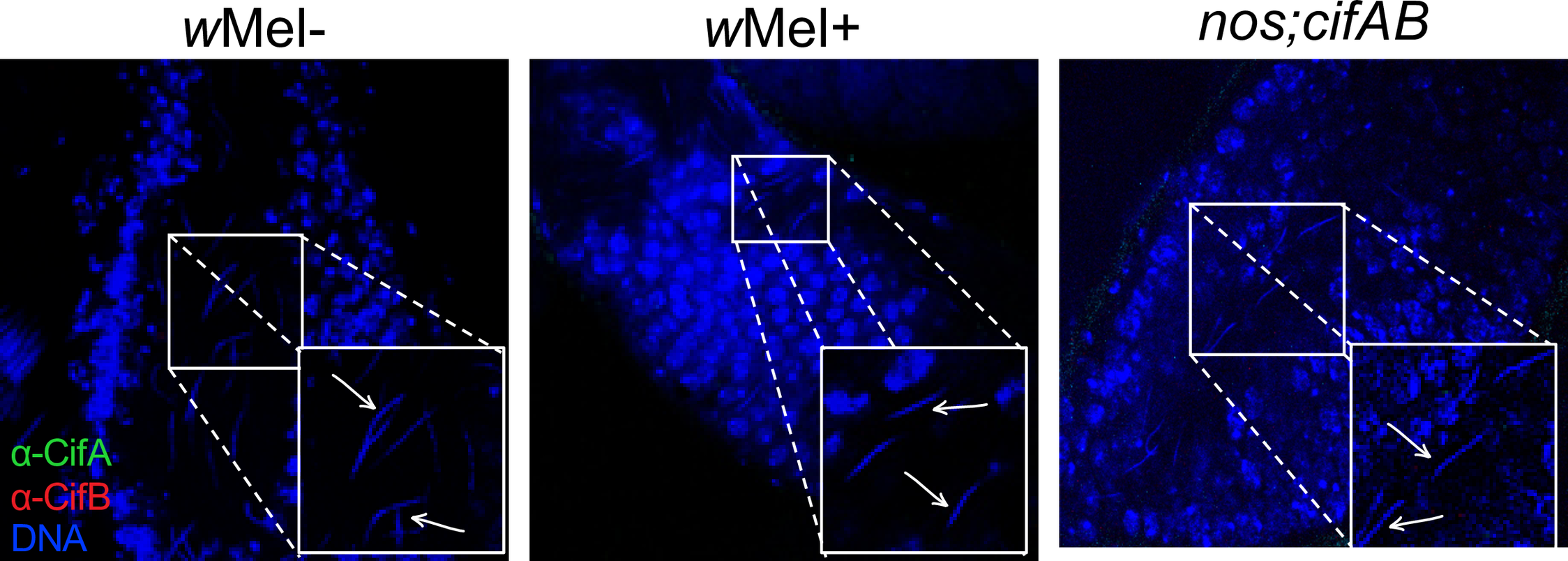

Supplement: S4 Fig — Seminal vesicles (n = 20) from <8-hour-old males of transgenic cifAB, wild-type wMel+ and wMel− lines were dissected and immunostained to visualize CifA (green) and CifB (red) in the mature condensed sperms (indicated by white arrows). DAPI stain (blue) was used to label nuclei. Absence of both CifA and CifB indicates that proteins are not accessible to the antibodies when the sperm chromatin is condensed and tightly packed. (TIF) [file pbio.3001584.s004.tif]

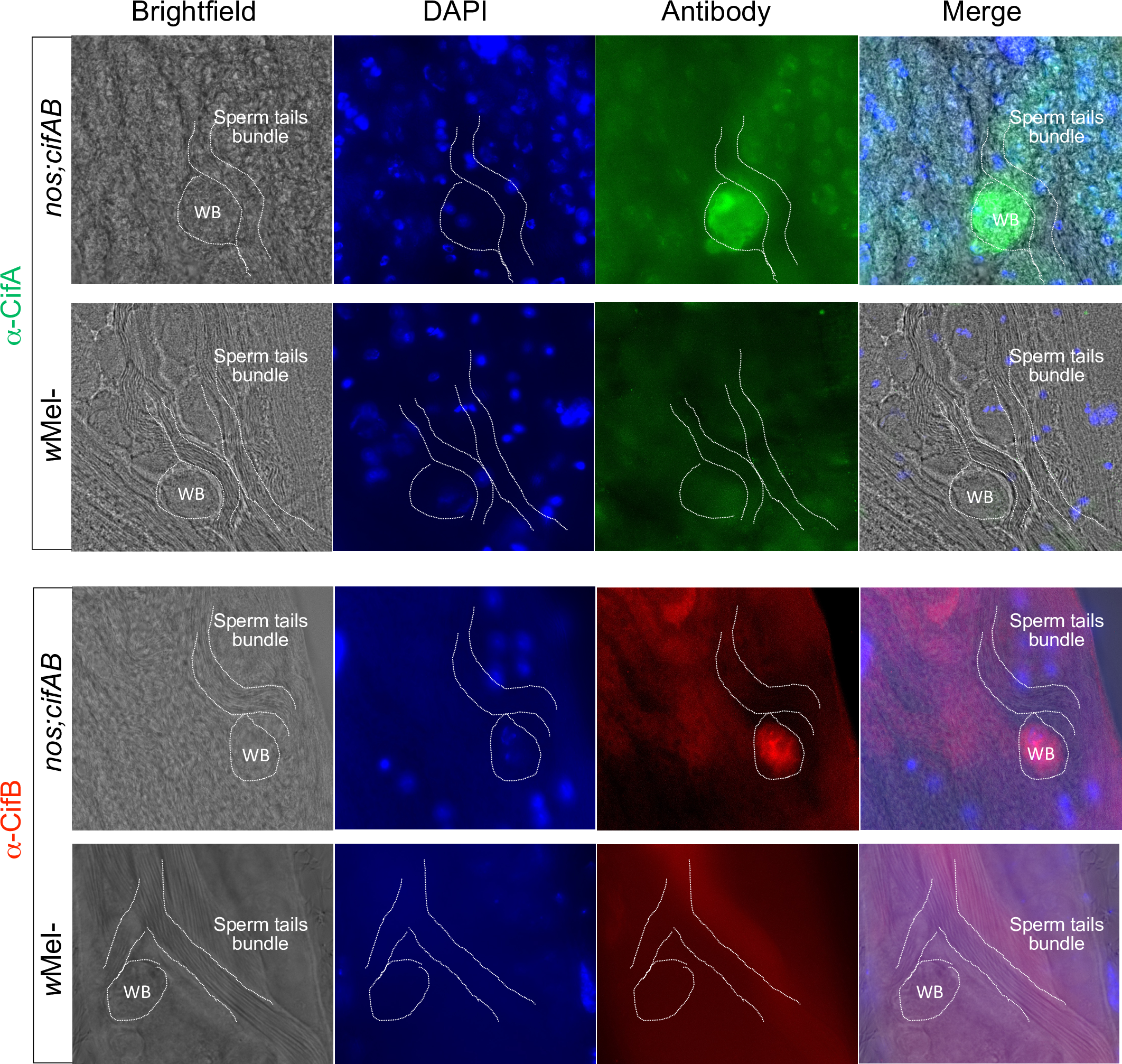

Supplement: S5 Fig — Testes (n = 20) from <8-hour-old males of transgenic cifAB, and wild-type wMel− lines were dissected and immunostained to visualize CifA (green) and CifB (red) in the cytoplasmic (WBs that are present near the basal end of sperm tail bundles. Some of the Cif proteins strip down in the WB in cifAB line and absent in wMel− control testes. Brightfield is shown to highlight the morphology of sperm tail bundles and WBs, which are otherwise not visible using Cif antibodies and DAPI stain. The experiment was run in parallel to the ones shown in Figs 1 and S2. WB, waste bag. (TIF) [file pbio.3001584.s005.tif]

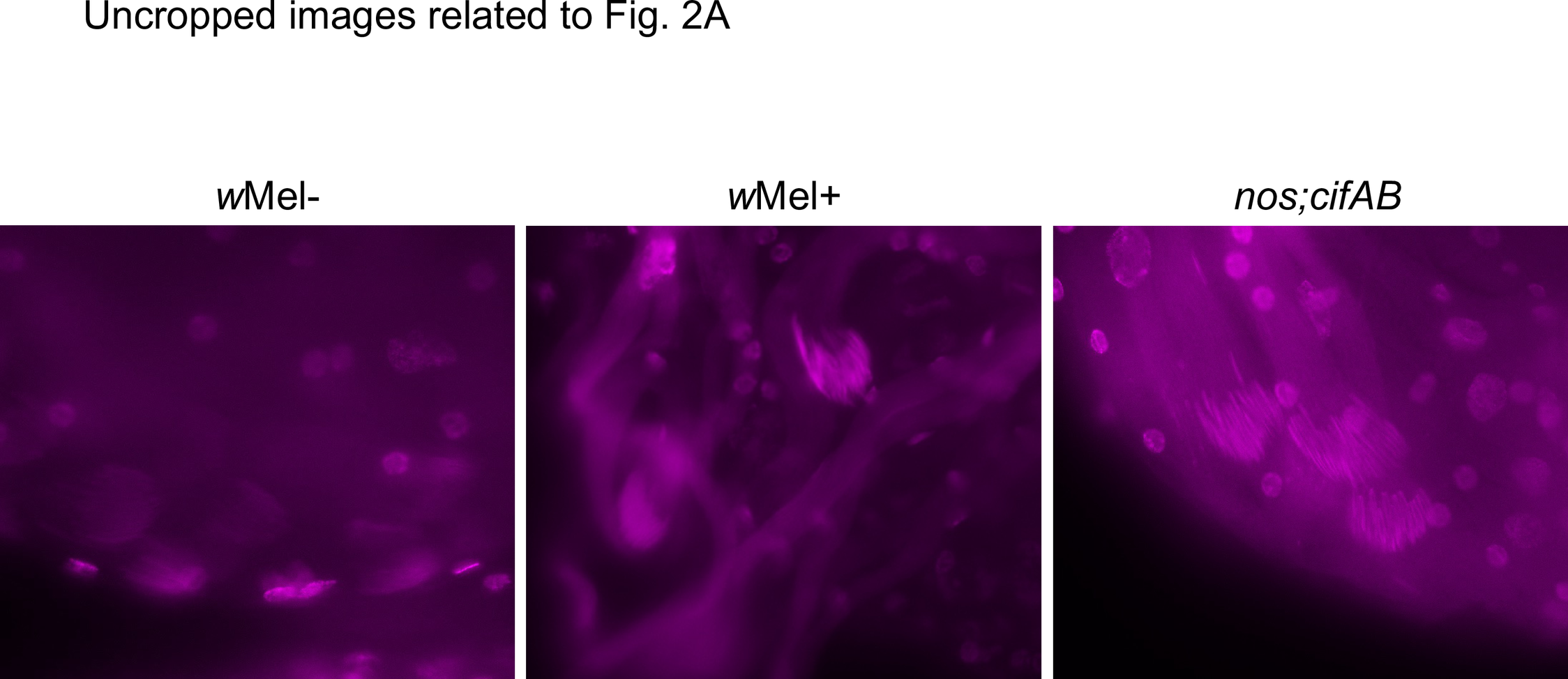

Supplement: S6 Fig — (TIF) [file pbio.3001584.s006.tif]

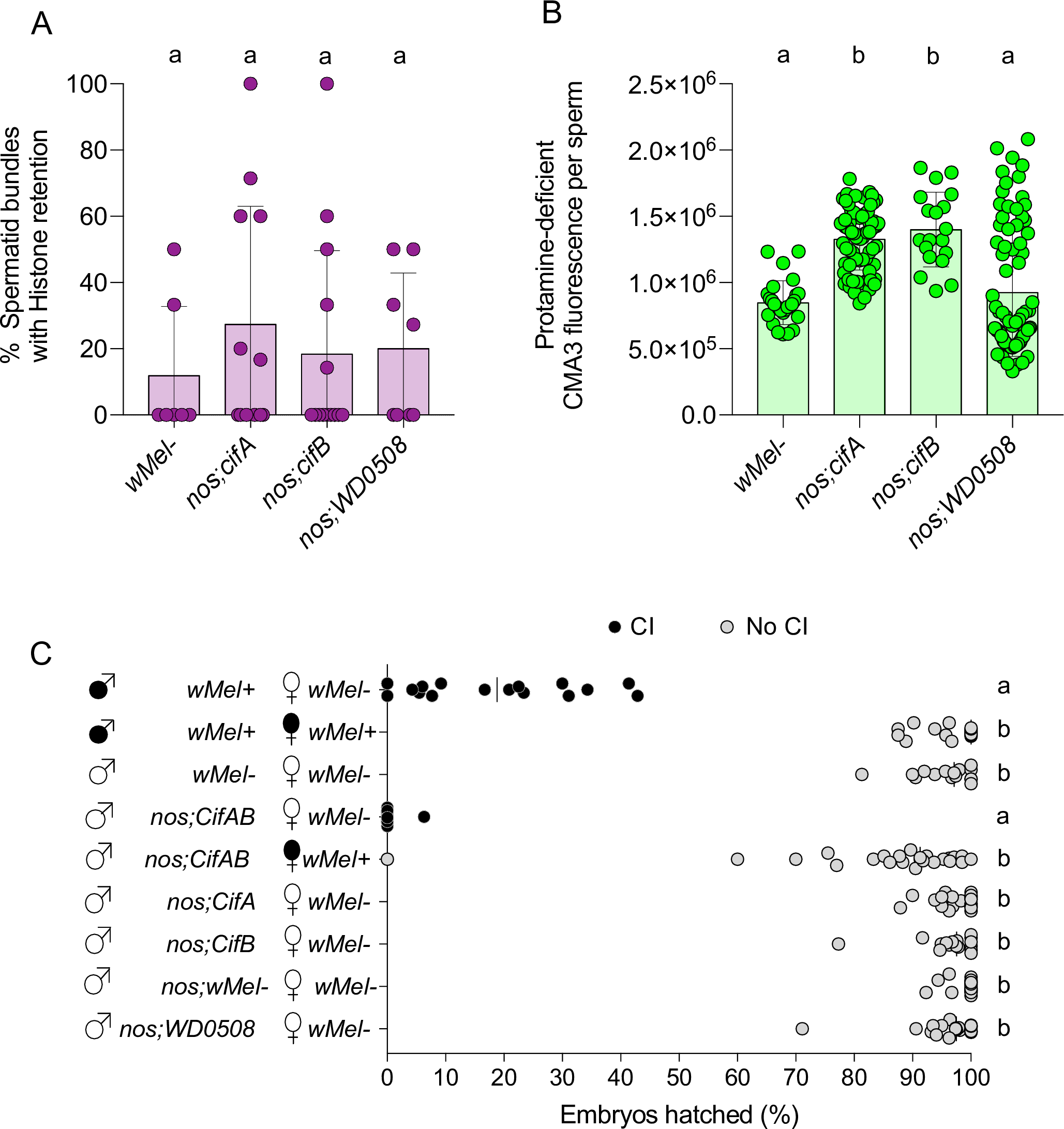

Supplement: S7 Fig — (A) Testes (n = 15) from <8-hour-old males of single transgene-expressing lines cifA, cifB, and a non CI-causing control gene WD0508 were dissected to quantify spermatid bundles with histone retention (purple) during late canoe stage of spermiogenesis. DAPI stain (blue) was used to label spermatid nuclei. Total spermatid bundles with DAPI signals and those with retained Histones were manually counted and graphed. Single transgenic expressing lines showed significantly less histones similar to the negative control wMel− at the late canoe stage. Vertical bars represent mean, and error bars represent standard deviation. Letters indicate statistically significant (p < 0.05) differences as determined by multiple comparisons based on a Kruskal–Wallis test and Dunn multiple test correction. (B) Mature sperms isolated from seminal vesicles (n = 15) of <8-hour-old males reared at 21°C were stained with fluorescent CMA3 (green) for detection of protamine deficiency in each individual sperm nucleus. Individual sperm head intensity was quantified in ImageJ (see Methods) and graphed. cifA- and cifB-expressing lines showed significantly higher fluorescence indicative of reduced levels of protamines compared to wMel− and WD0508 control lines. Vertical bars represent mean, and error bars represent standard deviation. Letters indicate statistically significant (p < 0.05) differences as determined by multiple comparisons based on a Kruskal–Wallis test and Dunn multiple test correction. All of the p-values are reported in S1 Table. The experiments were performed in parallel to the ones shown in Fig 2. (C) CI hatch rate analyses of transgenic male siblings used in CMA3 assays (Figs 2B and S6) validate that CI crosses (black circles) yielded significantly less embryonic hatching compared to non CI-inducing ones, when reared at 21°C. Letters to the right indicate statistically significant (p < 0.05) differences as determined by multiple comparisons based on a Kruskal–Wallis test and Dunn [file pbio.3001584.s007.tif]

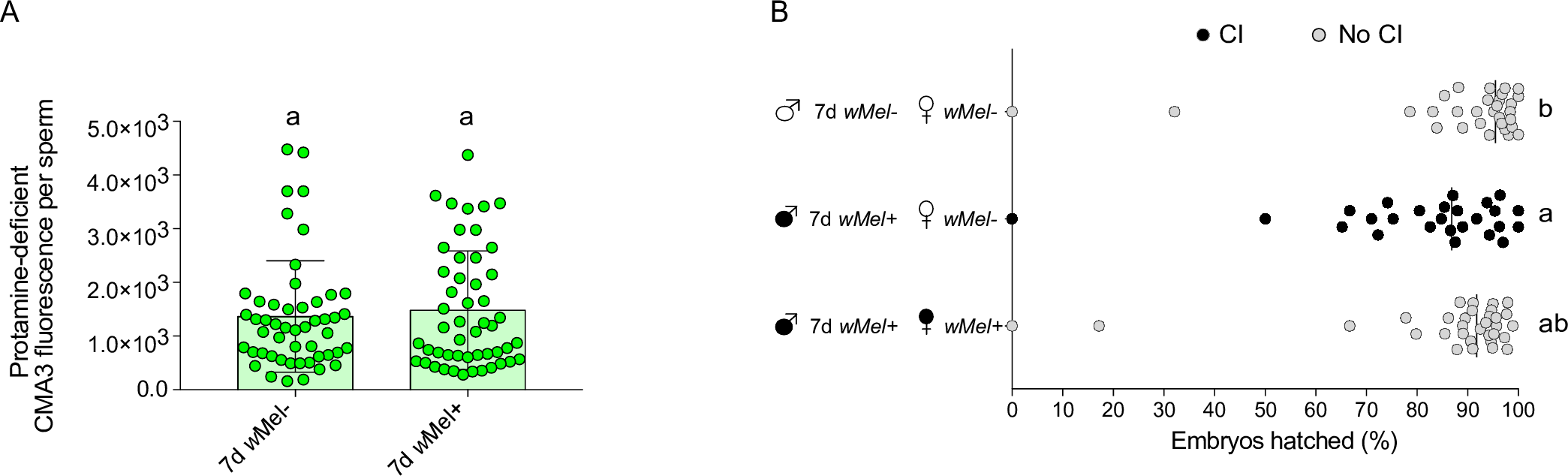

Supplement: S8 Fig — (A) Sperms from the 7-day-old wild-type Wolbachia-infected (wMel+) males show similar level of protamine levels as of wMel−. Vertical bars represent mean, and error bars represent standard deviation. Letters indicate statistically significant (p < 0.05) differences as determined by pairwise Mann–Whitney test. All of the p-values are reported in S1 Table. (B) CI hatch rate analyses of male siblings used in CMA3 assays (panel A) validate that 7d old wMel+ do not induce CI that correlates with their normal levels of sperm protamine levels. Letters to the right indicate statistically significant (p < 0.05) differences as determined by multiple comparisons calculated using a Kruskal–Wallis test and Dunn multiple test correction. All of the p-values are reported in S2 Table. Raw data underlying this figure can be found in S1 Data file. CI, cytoplasmic incompatibility; CMA3, chromomycin A3. (TIF) [file pbio.3001584.s008.tif]

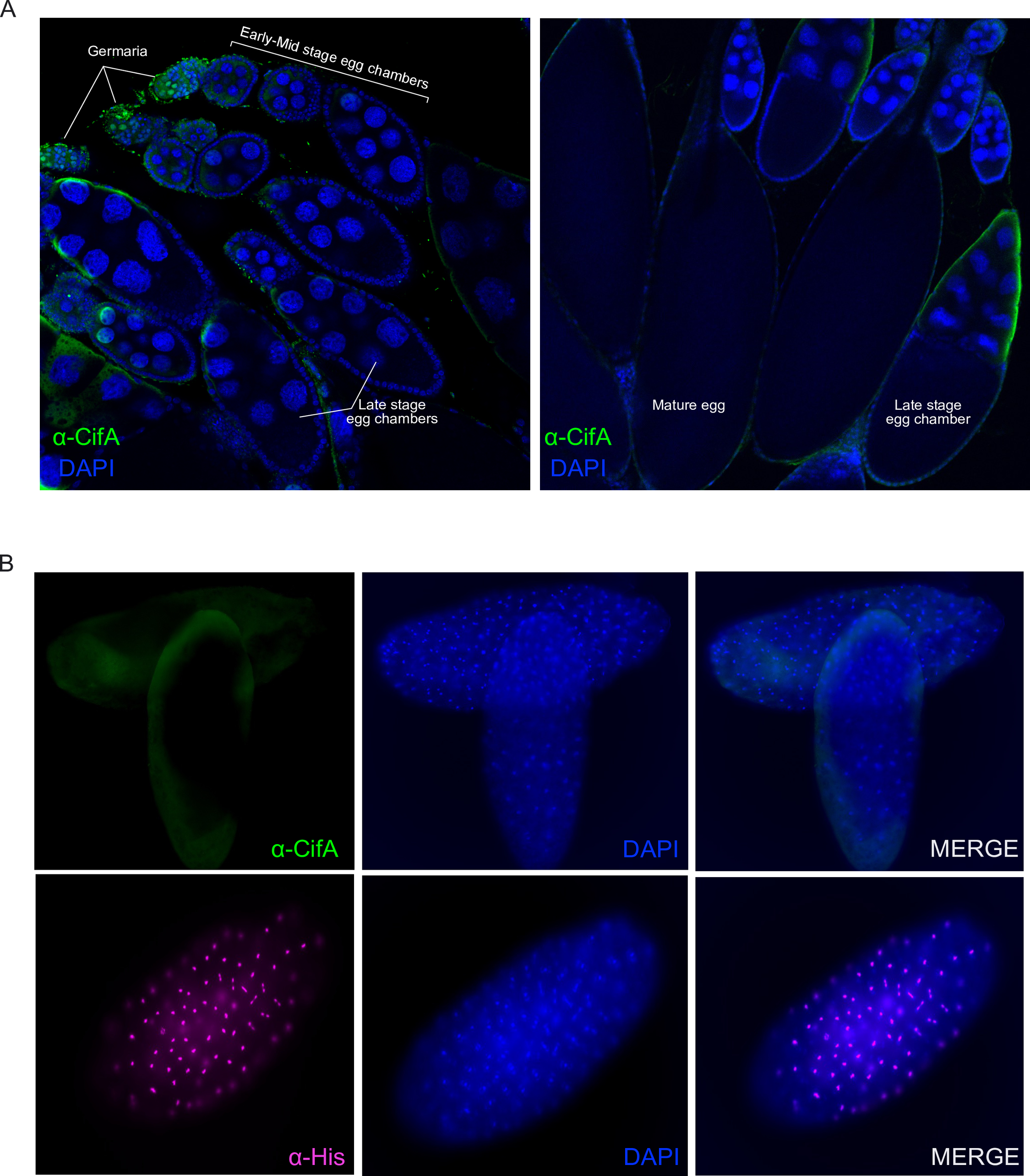

Supplement: S9 Fig — (A) In the transgenic cifA line, CifA (green) is absent in the late oocyte stages. Image was acquired at 20× magnification to show mid and late oocytes in one plane. We note the autofluorescence upon enhanced exposure in the green channel outlining the tissue morphology of stage 15 egg chamber does not signify CifA signals. (B) Immunofluorescence of CifA (green) and histones (magenta) in 1- to 2-hour-old embryos (n = 50) obtained from rescue cross (cifAB male × wMel+ female). Histone signals are detected in the developing embryos colocalizing with host DNA, labeled with DAPI (blue), whereas CifA signals are absent. (TIF) [file pbio.3001584.s009.tif]
