# Supplementary material for: The Cif proteins from Wolbachia prophage WO modify sperm genome integrity to establish cytoplasmic incompatibility
Source: PLoS Biol. 2022 May 24;20(5):e3001584. doi: 10.1371/journal.pbio.3001584 (PMC9128985; doi:10.1371/journal.pbio.3001584)
Supplement: S1 Raw Images — (DOCX) [file pbio.3001584.s015.docx]

S1 raw western blot image corresponding to Supplementary figure 1.
